# Mechanism of Bispecific Antibodies and Clinical Research Advances in Lung Cancer

**DOI:** 10.2174/0115680096353819250127065001

**Published:** 2025-06-12

**Authors:** Jie Yang, Yangyueying Liang, Meiying Zhu, Yuan Meng, Xuerui Wang, Minghui Yu, Longhui Li, Fanming Kong

**Affiliations:** 1 Department of Oncology, First Teaching Hospital of Tianjin University of Traditional Chinese Medicine, Tianjin, China;; 2 National Clinical Research Center for Chinese Medicine Acupuncture and Moxibustion, China;; 3 Tianjin Cancer Institute of Traditional Chinese Medicine, Tianjin, China

**Keywords:** Bispecific antibody, lung cancer, immunotherapy, targeted therapy, design strategies, mechanism of action

## Abstract

Lung cancer is an aggressive malignancy and one of the leading causes of cancer-related mortality worldwide. Compared with traditional treatments, the development of precision treatment programs, such as targeted therapy and immunotherapy, has progressively transformed non-small cell lung cancer (NSCLC) with driver mutations, becoming a clinically controllable chronic disease. Among the treatments for lung cancer, monospecific antibodies gradually show effectiveness but also expose their susceptibility to drug resistance, off-target effects, and other limitations. Therefore, bispecific antibodies have been developed, which have two different antigen-binding sites so that they can bind two distinct antigens or two distinct epitopes of the same antigen with adjustable specificity and do not easily produce drug resistance. This article reviews the design strategies and mechanism of bispecific antibodies, summarizes the latest progress in clinical trials involving bispecific antibodies for lung cancer, and analyzes the current challenges and future directions in this area of research.

## 1 INTRODUCTION

Lung cancer is a malignant tumor originating from respiratory epithelium, including bronchial tubes, small bronchioles, and alveoli. Lung cancer is categorized into two types based on histological lesions: NSCLC and small-cell lung cancer (SCLC), with NSCLC accounting for 80-85% of cases [1]. Although the global incidence of lung cancer is declining, NSCLC remains one of the leading causes of cancer-related mortality worldwide [2]. Cytotoxic chemotherapy, radiotherapy, and surgery have been the mainstay treatments for lung cancer. One of the major contributors contributing to disease recurrence is chemotherapy-induced multidrug resistance (MDR) [3]. However, with the advent of precision medicine, there has been an increased understanding of the immune microenvironment of lung cancer and the targetable oncogenic driver mutations. Immunotherapy and targeted therapy have significantly improved survival outcomes in patients with advanced lung cancer [4]. Compared to conventional chemotherapy, targeted therapy and immunotherapy are better targeting and less toxic, offering additional strategies for lung cancer treatment [5]. Bispecific antibodies, as a promising class in the development of next-generation precision therapy drugs, have been observed to be effective in hematologic tumors. In addition to half-life and safety, the immunosuppres-sive tumor microenvironment (TME) remains a major barrier to the use of bispecific antibodies in solid tumors. Currently, more than 100 clinical trials involving bispecific antibodies for multiple solid malignancies have yielded encouraging preclinical results [6, 7].

Therapeutic use of monoclonal antibodies emerged after Kohler and Milstein proposed the hybridoma technique for the production of monoclonal antibodies in 1975 [8]. Monoclonal antibodies are employed extensively in the clinical treatment of cancers because they are more selective, effective, and have fewer adverse effects than traditional chemotherapies [9]. However, clinical experience has revealed that monoclonal antibodies have limited efficacy and may lead to tumor resistance and off-target effects [10]. Given the complexity of cancer biology, targeting multiple pathways simultaneously holds significant promise, which has led to the development of bispecific antibodies [11, 12]. Bispecific antibodies, through their two different antigen-binding sites, can simultaneously recognize and bind to two different epitopes on tumor cells, thus enhancing the targeting of tumor cells [13]. The advancement of bispecific antibody production platforms has been greatly facilitated by the development of genetic engineering technology, leading to an increasing number of bispecific antibodies entering clinical trials through technological innovation [14, 15]. Bispecific antibodies are distinct from traditional monoclonal antibody therapies or combinations of monoclonal antibodies. They overcome the limitations of monoclonal antibodies, exhibiting higher sensitivity and binding specificity.

Furthermore, compared to combination therapies involving two monospecific antibodies, bispecific antibodies can avoid the development of drug resistance and reduce costs [16, 17]. With the appropriate combination of targets and optimized structural design, bispecific antibodies can achieve the desired therapeutic effects and be produced at a large scale. This review aims to summarize the design strategies, mechanisms of action, and current research status on bispecific antibodies in the treatment of lung cancer.

## 2 DESIGN STRATEGIES AND MECHANISM OF ACTION

### 2.1 Design Strategies

Bispecific antibodies with precisely defined specificities are rare in nature. Most of them must be artificially synthesized or adapted from natural antigens [18]. Currently, bispecific antibodies are primarily produced by three methods: cellular hybridoma technology, chemical coupling methods, and recombinant DNA methods [19]. These antibodies can be classified into two main categories based on various design strategies and the presence or absence of the fragment crystallizable (Fc) region: Fc-containing bispecific antibodies and Fc-lacking bispecific antibodies.

**Fig. (1):**
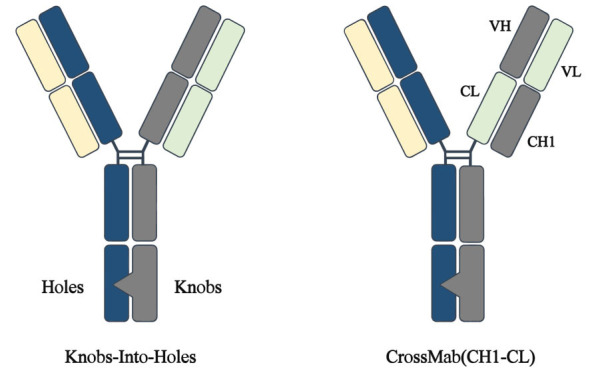
Schematic representation of the two main bispecific antibody design strategies.

Fc-containing bispecific antibodies share structural similarities with natural antibodies, earning them the name “Immunoglobulin G (IgG)-like structure bispecific antibodies” [20]. The large molecular size of IgG-like bispecific antibodies aids in their purification, enhances stability and solubility, prolongs serum half-life, increases affinity, and consequently boosts biological activity. These properties might lead to potential advantages in terms of administration frequency. With the presence of an Fc region, they can induce Fc-mediated effector functions, such as Antibody-Dependent Cell-Mediated Cytotoxicity [21]. Moreover, the activity of the Fc segment can be modulated based on functional requirements. Nonetheless, bispecific antibodies containing Fc regions possess higher molecular weights, limited penetration into tumor tissues, complex production processes, and increased costs. Additionally, the Fc region can trigger immune response, and immunotherapy utilizing bispecific antibodies targeting T cells may potentially lead to uncontrolled systemic inflammatory reactions [22]. Fc-containing bispecific antibodies can be further categorized into symmetric and asymmetric structures based on symmetry. Symmetric bispecific antibodies can overcome challenges related to large-scale production and purification, facilitate the preparation of homogeneous bispecific antibody drugs, and improve the reproducibility and stability of production and stability of production. Notable examples include the “Two-in-One” technology. The DAF (Two-in-One) technology was first proposed by Genentech (California, USA), and applies phage display library technology to reshape the binding surface of the antibodies, enabling high affinity for binding to a second antigen, that is, the same binding site of the antibody can be modified to bind two different antigens with high affinity. This technology retains the structure of normal IgG antibodies, maintaining its stability, and can be produced using existing universal antibody technologies [23]. Duligotuzumab is a bacteriophage-derived DAF humanized antibody. It binds to epidermal growth factor receptor (EGFR) and human epidermal growth factor receptor 3 (HER3), inhibiting downstream signaling pathways and suppressing tumor cell growth [24]. Asymmetric IgG-like bispecific antibodies consist of two distinct heavy chains originating from antibodies targeting different antigens. To create the desired IgG-like bispecific antibody, two primary issues need to be resolved: the separation of the light-chain/heavy-chain interactions and the heterodimerization of two different heavy chains [25]. Various innovative techniques, like “Knobs-Into-Holes”, “CrossMab” and “Duobody” platforms, ensure that dissimilar heavy and light chains are paired correctly. In the “Knob-In-Hole” approach, a larger amino acid is substituted for a smaller one in the CH3 domain of one chain to form a “knob” structure, while in the other chain, a smaller amino acid is substituted for a larger one to form a “hole” structure. The complementary spatial structure of these “knob” and “hole” regions enables the accurate assembly of the two original antibodies [13]. The “Knobs-Into-Holes” strategy not only facilitates the creation of antibody-immunoadhesin hybrids but also holds the potential for developing other Fc-containing bispecific therapeutics [13, 26]. The “CrossMab” technology, an extension of “Knob-In-Hole” technology, alters the molecular structure of the interface between VH-VL and CH1-CL regions by reengineering the heavy and light chains of the Fab region of one of the antibodies. This study illustrates the similarities and differences between the “CrossMab” and “Knob-In-Hole” design strategies in Fig. **1**. The representative products developed using this platform include the vascular endothelial growth factor(VEGF)×Ang-2 bispecific antibodies, RG7221 and RG7716 [27]. The “Duobody” platform aids in completing an exchange of fab arms between two antibodies through the introduction of K409R and F405L mutation sites in the CH3 region, resulting in the formation of a bispecific antibody. Amivantamab, an anti-EGFR×mesenchymal-epithelial transition factor (MET) bispecific antibody developed for NSCLC using this platform, has received regulatory approval [28].

“Knob-In-Hole” and “CrossMab,” highlighting their structural differences.

Non-IgG-like bispecific antibodies, due to the absence of Fc regions, exhibit higher tumor tissue permeability, resulting from their lower molecular weight and greater tumor specificity. However, they are also more prone to degradation by lysosomal enzymes in target cells. Most of these antibodies have a short half-life and require multiple administrations [29]. Targeted killing of cancer cells is achieved by bispecific T cell engager (BiTE), which links the CD3 antibody single-chain variable fragment (ScFv) with the tumor-associated antigen (TAA) ScFv using the G4S linker [30]. Tarlatamab (AMG 757), developed using the BiTE platform, connects both CD3 and delta-like canonical Notch ligand 3 (DLL3), mediating tumor cell lysis through T-cell activation [31].

### 2.2 Mechanism of Action

Bispecific antibodies can exert multiple mechanisms of action. Some bispecific antibodies bridge immune cells and tumor cells, thereby enabling the immune system to target and eliminate tumor cells. Bispecific antibodies currently under development globally are mainly based on T-cell redirection, with a particular focus on CD3-based bispecific antibodies [32]. Other bispecific antibodies act on dual-target immune checkpoints and competitively bind to immune checkpoints, enhancing immune responses. Research on bispecific antibodies in China mainly focused on dual immune checkpoint blockade, especially on dual antibodies targeting the programmed cell death protein 1 (PD-1) and programmed cell death-ligand 1 (PD-L1) pathway [33].

Bispecific antibodies specifically recognize tumor cells and eliminate them by immune cells through the cellular bridging mechanism. This connection enables redirected effector T cells to exert a cytotoxic effect, leading to the elimination of tumor cells [32]. In recent years, T-cell bispecific antibodies, especially those targeting CD3-positive T cells, have gained significant attention in research. CD3 molecules can form the T-cell receptors (TCR)-CD3 complex with the TCR on T-cell surfaces [34]. This complex, which functions independently of major histocompatibility complex (MHC) restrictions, acts as the primary signal for T-cell activation along with CD28 co-stimulation, inducing T-cell activation irrespective of TCR epitope specificity [35]. Upon binding to bispecific antibodies, T cells form an immune synapse with target cells. This synapse triggers the release of high levels of granzyme and perforin, which are responsible for tumor cell lysis [36]. Moreover, synapse formation leads to TCR crosslinking, prompting T cells to release cytokines that promote inflammation and stimulate T-cell expansion [37]. However, a serious adverse effect called cytokine release syndrome (CRS) can result from excessive immune activation. One of the major challenges in developing this type of bispecific antibody is accurately distinguishing between normal and tumor cells [38]. The two approved bispecific antibodies, catumaxomab, and blinatumomab, both function through this mechanism [20]. T-cell redirected bispecific antibodies are also being explored for the treatment of solid tumors.

Currently, treatment with immune checkpoint inhibitors (ICIs) is becoming increasingly prevalent. Immune checkpoints, as immunosuppressive pathways, play a pivotal role in upholding autoimmune tolerance and modulating immune responses in peripheral tissues [39]. However, these pathways can become aberrantly and persistently activated by tumors, which suppresses anti-tumor immunity and promotes tumorigenesis [40]. In recent years, PD-1 and PD-L1 have emerged as prominent targets in clinical trials for NSCLC [41]. Furthermore, several novel immune checkpoints, including LAG3, TIM3, TIGIT, and BTLA, have been identified in recent studies. Building on these immune checkpoints, a series of clinical trials have been devised [42]. Simultaneously, targeting immune checkpoints expressed by both tumor and immune cells, while alleviating tumor-mediated immunosuppressive signaling pathways, can mitigate immune evasion of tumor cells and enhance immune-mediated tumor cell killing, all while minimizing detrimental effects on normal cells and improving treatment safety [43].

By changing their signaling pathways as a countermeasure against single signaling blockage, tumor cells can escape. Bispecific antibodies, which target two TAA simultaneously, can disrupt dual signaling pathways associated with tumor growth, thereby overcoming the resistance mechanism. Amivantamab, which utilizes this dual-target mechanism, has accelerated the approval process by the U.S. Food and Drug Administration (FDA) as the first treatment for EGFR exon 20insertion (exon20ins) mutations [44]. Furthermore, bispecific antibodies, such as SI-B001 and duligotuzumab, which target EGFR and HER3, are currently in the developmental phase [45, 46]. There are also bispecific antibodies that enhance immune cell-mediated tumor killing by simultaneously blocking immune checkpoints and blocking tumor-promoting pathways in the TME. Engineered to inhibit PD-L1 and transforming growth factor β (TGF-β) on tumor cells, SHR-1701 can rejuvenate and improve anti-tumor immune responses [47]. Another class of bispecific antibodies targets two non-overlapping epitopes of the same antigen. These antibodies bind to two distinct epitopes, simultaneously binding to both epitopes on the antigen and facilitating the formation of aggregated or cross-linked complexes. This aggregation and cross-linking increase the binding strength and affinity between the antibody and antigen, thereby enhancing the binding efficacy of the antibody.

**Fig. (2):**
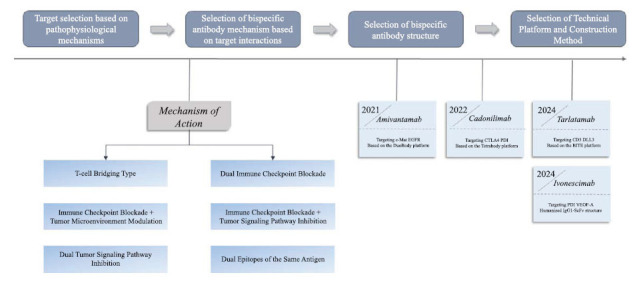
Development process, mechanism of action, and approved bispecific antibody therapies for solid Tumors.

Furthermore, dual epitope binding promotes internalization, leading to the uptake of the antibody-antigen complex by the cell. This is especially crucial for therapeutic strategies that rely on internalization to mediate their effects. Additionally, dual epitope binding can enhance the Fc-mediated immune effects of the antibody, thereby amplifying the overall immune response [48, 49]. This increased affinity results in a significant rise in tumor uptake of bispecific antibodies within 72 hours and improved tumor localization within 48 hours after injection [50]. Currently, the bispecific antibody KN026, which targets two non-overlapping HER2 epitopes, is under clinical investigation. KN026 integrates anti-HER2 Fc-based heterodimeric molecules derived from trastuzumab and pertuzumab with similar affinities. However, it shows enhanced tumor cell binding compared to its parental monoclonal antibodies [51]. Additionally, bispecific antibodies can be engineered to target TAAs and immunomodulatory receptors, aiming to enhance the efficacy of immunotherapy by disrupting immunosuppression signals, activating immune responses, and promoting tumor cell destruction. This study presents the development process and mechanism of action of bispecific antibodies in the form of a flowchart in Fig. **2**.

## 3 TARGETED BISPECIFIC ANTIBODIES

### 3.1 Bispecific Antibodies Targeting EGFR and MET

Amivantamab is a bispecific antibody that targets both the EGFR and MET receptors. This fully human antibody belongs to the IgG1 class, exhibiting Fc activity. It has the unique ability to inhibit both EGFR and a common resistance mechanism to EGFR-targeted treatment *via* the MET pathway simultaneously. The dual inhibition holds promise for augmenting the effectiveness and longevity of treatment responses in patients harboring these mutations [52, 53]. The CHRYSALIS study is a phase I, open-label, dose-escalation, and dose-expansion study, which included a population with EGFR exon20ins and MET exon 14 (MET14) skipping mutations NSCLC. The primary endpoint is the overall response rate (ORR), and key secondary endpoints include overall survival (OS), duration of response (DOR), progression-free survival (PFS), and clinical benefit rate (CBR). The EGFR exon20ins population included a total of 81 patients, all of whom had previously undergone platinum-based treatment as part of their prior therapies. The study found that amivantamab showed promising anti-tumor activity and durable response in NSCLC patients with EGFR exon20ins who failed platinum-based chemotherapy with an ORR of 40% (95% confidence interval (CI): 29%-51%), a median DOR of 11.1 months (95% CI: 6.9 to not reached), a CBR of 74% (95% CI: 63%-83%), a median PFS of 8.3 months (95% CI: 6.5-10.9), and a median OS of 22.8 months (95% CI: 14.6 to not reached) [54]. On May 21, 2021, the FDA granted accelerated approval for the treatment of patients with NSCLC harboring EGFR Exon20ins mutations who had failed platinum-based chemotherapy, based on the results of this study [28]. The most frequent adverse events (AEs) based on the therapy cohort included paronychia (45%), infusion-related reaction (66%), and rash (86%). Grade 3 or higher treatment-related AEs occurred in 16% of patients, with the most common being neutropenia (3%), infusion-related reactions (3%), and rash (4%) [54]. As of February 22, 2023, a total of 97 patients with primary MET14 mutations were evaluated. In the overall population, the ORR was 33%. The DOR in the responders was 11.2 (95% CI: 4.3-19.1) months. Amivantamab treatment demonstrated durable clinical benefit, with the longest response lasting 29 months [55]. The study also found that in patients with progressing EGFR-mutated NSCLC who had previously received osimertinib and platinum-based chemotherapy, the amivantamab and lazertinib combination therapy showed good tolerability and efficacy. Among 50 efficacy-evaluable patients, the ORR was 36% (95% CI: 23%-51%), and the CBR was 58% (95% CI: 43%-72%). The anti-tumor activity was comparable to previously reported results in the post-lazertinib resistance, chemotherapy-primed population, suggesting that chemotherapy may not affect the activity of amivantamab and lazertinib [56]. For relapsed or refractory EGFR-mutated NSCLC, amivantamab in combination with lazertinib and platinum-based chemotherapy, resulted in an ORR of up to 50% (95% CI: 27%-73%) in all patients. The results of the study suggest that the combination of amivantamab, lazertinib, and chemotherapy is highly effective in patients with EGFR tyrosine kinase inhibitor (TKI)-resistant disease. Patients with brain metastases at baseline had the same ORR and CBR. With no warning signs, the safety of the LACP regimen was in line with that of single medications [57]. Most of the AEs were controllable with dose modifications or standard medical interventions [58]. In the phase III international randomized MARIPOSA study (NCT04487080), the median PFS was markedly extended in the amivantamab–lazertinib cohort compared to the osimertinib cohort (23.7 vs. 16.6 months). This trial demonstrated that the combination of amivantamab and lazertinib offers greater efficacy than osimertinib as first-line therapy in advanced NSCLC with EGFR mutations. Based on the results of this study, the FDA has approved the combination of amivantamab and lazertinib for the first-line treatment of adult patients with locally advanced or metastatic NSCLC with EGFR exon 19 deletions or exon 21 L858R substitution mutations [59]. At the 2024 European Society for Medical Oncology (ESMO) congress, investigators presented a comparison of acquired resistance mechanisms in the phase III MARIPOSA study between amivantamab plus lazertinib and osimertinib as first-line treatments for advanced NSCLC. Relative to the osimertinib group, the amivantamab plus lazertinib group exhibited a significantly lower incidence of acquired MET amplification (4.4% vs. 13.6%; P=0.017) and EGFR resistance mutations, including C797S, L718X, and G724X (0.9% vs. 7.9%; P=0.014). Additionally, the incidence of acquired TP53 resistance mutations was lower in the amivantamab plus lazertinib group (9.7% vs. 12.9%). These preliminary results suggest that first-line treatment with amivantamab plus lazertinib effectively suppresses resistance associated with EGFR and MET pathway alterations [60]. A multicenter, open-label phase I study (NCT04667975) evaluating CKD-702 demonstrated a manageable safety profile regarding EGFR and MET inhibition, with initial responses observed in MET14 patients. Therefore, more clinical studies are needed to determine the clinical benefit of CKD-702 in NSCLC patients [61].

### 3.2 Bispecific Antibody Targeting MET and MET

A human bispecific MET antibody called REGN5093 causes MET to internalize and degrade. Without triggering MET-driven biological reactions, REGN5093 blocks MET-mediated signaling and stops the proliferation of tumor cells driven by MET [62]. A multicenter, open-label, first-in-human Phase I/II study (NCT04077099) is investigating the safety and efficacy of REGN5093 in MET-altered advanced NSCLC patients [63]. The safety results indicated no evidence of dose-limiting toxicity [64]. REGN5093 monotherapy for MET-mutated advanced NSCLC patients who have undergone multiple prior treatment lines has shown safety and tolerability, and preliminary efficacy signals were observed in patients with MET gene amplification and MET14 exon skipping mutation, and MET protein over-expression [65].

### 3.3 Bispecific Antibody Targeting HER2 and HER3

A multicenter, open-label Phase I/II clinical trial (NCT02912949) is evaluating the safety, tolerability, pharmacokinetics (PK), pharmacodynamics (PD), immunogenicity, and antitumor activity of zenocutuzumab (MCLA-128), targeting HER2 and HER3 in patients with solid tumors harboring neuregulin-1 (NRG1) fusions. In solid tumors, NRG1 rearrangements are recurrent oncogenic drivers. When NRG1 binds to HER3, more downstream signaling, tumorigenesis, and heterodimerization with other HER/ERBB kinases result. Zenocutuzumab suppressed HER3 and AKT activation, induced apoptosis marker expression, and restrained growth [66, 67]. The confirmed ORR was 34% (90% CI: 25%-44%) among 71 patients with measurable disease, which included responses in NSCLC, pancreas cancer, breast cancer, and cholangiocarcinoma [68]. Zenocutuzumab demonstrated anti-tumor effects and durable responses in NRG1-positive tumor patients. The FDA has placed priority review on biologics license application (BLA) seeking approval of zenocutuzumab for the treatment of patients with NRG1-positive NSCLC and pancreatic cancer [66]. However, the study also faces several challenges, including the rarity and heterogeneity of NRG1 fusions, the lack of a validated diagnostic test, the potential resistance mechanisms, and the optimal combination strategy. Therefore, more trials are required to establish the clinical utility and benefit of zenocutuzumab for patients with NRG1-positive tumors [66].

There are still phase III clinical trials of amivantamab underway with a maximum sample size of up to 1,074 cases [69-72]. Phase III clinical studies of targeted bispecific antibodies in lung cancer are presented in Table **1**.

## 4 IMMUNE BISPECIFIC ANTIBODIES

### 4.1 Bispecific Antibodies Targeting PD-L1 and TGF-β

Bintrafusp alfa is a bispecific antibody designed to colocalize and simultaneously inhibit two immunosuppressive pathways in the TME: TGF-β and PD-L1 [73]. Advanced NSCLC patients who either developed resistance to anti-PD-L1 treatment or progressed after chemotherapy were included in this expansion cohort of a global, open-label, phase I trial (NCT02517398). Every two weeks, they received intravenous bintrafusp alfa 500mg (n=40) or 1200mg (n=40) until disease progression, intolerable toxicity, or withdrawal from the study [73, 74]. The trial reported that bintrafusp alfa had linear pharmacokinetics and showed encouraging clinical activity. The median OS for the entire cohort was 13.6 months (95% CI: 10.9-19.3). For the 500-mg dose group, the median OS was 10.9 months (95% CI: 4.5-16.0), while the 1200-mg dose group had a median OS of 17.1 months (95% CI: 12.0-26.7). The incidence of AEs and immune-related AEs was 68.8% and 17.5%, respectively [75]. These results suggest a potential survival benefit of bintrafusp alfa over other second-line therapies for NSCLC [76]. SHR-1701 showed high PD-L1 target occupancy and a strong affinity for TGF-β1, TGF-β2, and TGF-β3 [77]. A phase I study (NCT03774979) was conducted to assess SHR-1701 in 27 patients with advanced solid tumors. The trial indicated that the drug was safe and well-tolerated. It demonstrated promising anti-tumor activity in EGFR-positive NSCLC patients who were resistant to EGFR TKI treatment. The ORR was 16.7% (95% CI: 4.7%-37.4%), and the DCR was 50.0% (95% CI: 29.1%-70.9%). However, the trial had several limitations, such as a small participant pool and a short follow-up duration [78, 79]. In a subgroup analysis of PD-L1 positive advanced or metastatic NSCLC, the ORR was 52.0% in patients with a PD-L1 tumor proportion score (TPS) ≥50%, compared to 37.0% in those with TPS <50%, which is a significant improvement from the reported ICIs monotherapy, and SHR-1701 had a superior ORR in patients having PD-L1 TPS ≥50%. Subsequently, in a phase II trial of induction therapy with combination chemotherapy for stage III unresectable NSCLC, SHR-1701 combined with chemotherapy induction after surgery or radiotherapy showed good anti-tumor activity and was safe and manageable. About 25.2% of patients were converted from inoperable to operable after receiving induction therapy, and this data supports the subsequent phase III trial [80]. In a subsequent trial, the combination of neoadjuvant SHR-1701 with chemotherapy, followed by either surgery or radiotherapy, demonstrated encouraging effectiveness and an acceptable safety profile in 97 patients with unresectable stage III NSCLC. Of these patients, 27 (25%) proceeded to surgery, with all achieving R0 resection. Among these surgical patients, 12 (44%) exhibited major pathological responses, while seven (26%) achieved pathological complete responses [81]. A Phase III clinical trial presented at the 2024 ESMO congress evaluated the efficacy of SHR-1701 in combination with chemotherapy as first-line treatment for HER2-negative gastric/gastroesophageal junction adenocarcinoma. In the intention-to-treat population, median OS was significantly longer with SHR-1701 plus chemotherapy compared to placebo plus chemotherapy (15.8 vs. 11.2 months; HR 0.66, 95% CI: 0.53–0.81; P<0.0001). SHR-1701 thus became the first and currently the only bispecific antibody to achieve positive results in this patient population [82]. Additionally, SHR-1701, in combination with XELOX and bevacizumab, showed manageable safety and effective antitumor activity in patients with unresectable metastatic colorectal cancer, with an ORR of 59.7% (95% CI: 47.3%–71.0%) and a DCR of 83.9% (95% CI: 72.8%–91.0%) [83]. Furthermore, SHR-1701 is currently being investigated in Phase III trials for first-line treatment of cervical cancer and other indications [84].

### 4.2 Bispecific Antibodies Targeting PD-1/PD-L1×CTLA-4

Bispecific IgG-ScFv antibody cadonilimab (AK104) binds to both cytotoxic T lymphocyte antigen 4 (CTLA-4) and PD-1 [85]. A single arm, two cohorts, phase Ib/II trial (NCT04646330) examined AK104 with anlotinib in advanced NSCLC patients [86]. AK104 plus anlotinib is a novel combination therapy that targets both immune checkpoints and tumor angiogenesis. The trial results showed that among 69 patients receiving cadonilimab in combination with anlotinib, 37 achieved confirmed partial responses (PR), resulting in an ORR of 53.6% (95% CI: 41.2%-65.7%) and a DCR of 92.8% (95% CI: 83.9%-97.6%). The ORR for PD-L1-positive and PD-L1-negative patients was 60.5% (95% CI: 44.4%-75.0%) and 42.3% (95% CI: 23.4%-63.1%), respectively. In this study, the cadonilimab and anlotinib combination regimen demonstrated promising efficacy and manageable safety in the first-line treatment of NSCLC. This offers a novel chemotherapy-free combination strategy using a bispecific antibody and an anti-angiogenesis agent for the first-line treatment of advanced NSCLC [87]. Cadonilimab has demonstrated excellent efficacy and safety across various malignancies, supporting its potential use as a monotherapy or combination therapy for patients with advanced solid tumors. The phase III COMPASSION-16 trial evaluated the efficacy of adding cadonilimab to first-line standard chemotherapy in patients with persistent, recurrent, or metastatic cervical cancer. The results showed a median PFS of 12.7 months (95% CI: 11.6–16.1) in the cadonilimab group, compared to 8.1 months (95% CI: 7.7–9.6) in the placebo group [88]. These data support the use of cadonilimab in combination with chemotherapy as an effective first-line treatment for persistent, recurrent, or metastatic cervical cancer. Additionally, clinical trials assessing the efficacy of cadonilimab in hepatocellular carcinoma (NCT04728321) and gastric adenocarcinoma (NCT05008783) are ongoing. Another antibody targeting PD-1 and CTLA-4, called MEDI5752, is being investigated for its potential to treat a range of advanced cancers. Preliminary results showed that combination chemotherapy with MEDI5752 as the first line of treatment for non-squamous NSCLC is numerically superior to pembrolizumab combination chemotherapy in terms of median OS and median PFS. This improvement suggests a potential option for a first-line standard of care, especially for patients with PD-L1 expression <1% [89]. KN046 is a bispecific antibody that simultaneously binds to both PD-L1 and CTLA-4. This prevents PD-L1 from binding to PD-1 and CTLA-4 from binding to CD80/CD86. Compared to CTLA-4, KN046 binds PD-L1 more firmly and exerts a stronger inhibitory effect on cancers with high PD-L1 expression [90, 91]. The KN046-201 (NCT03838848) trial is a multicenter, open-label, single-arm study, including various cohorts to assess the safety, tolerability, and efficacy of KN046 in 64 patients with advanced NSCLC. The ORR for all 64 participants was 14.1% (95% CI: 6.64%-25.02%), with a median PFS of 3.7 months (95% CI: 2.9-5.5), and a median OS of 18.4 months (95% CI: 12.9-21.9). Furthermore, the ORR for non-squamous NSCLC was 17.1% (95% CI: 7.15%-32.06%), while for squamous NSCLC, the ORR was 10.0% (95% CI: 1.23%-31.70%). The findings demonstrated that KN046 is effective in treating patients with advanced NSCLC who were both squamous and non-squamous, had not responded to first-line platinum-based chemotherapy, and had not previously received immunotherapy. However, the high incidence of grade 3 or higher levels of AEs (42.2%) with this agent alone suggests that this therapy may have significant side effects, potentially limiting its use in certain patients [92, 93]. Additionally, a phase II trial (NCT04054531) demonstrates that platinum-based chemotherapy combined with first-line KN046 is a promising therapeutic option for patients with metastatic NSCLC. This combination is also effective and well-tolerated in these patients.

### 4.3 Bispecific Antibody Targeting PD-1/PD-L1×VEGF

Ivonescimab (AK112) is a bispecific antibody that simultaneously targets PD-1 and VEGF. Patients with advanced NSCLC who had progressed after primary or first-line platinum-based chemotherapy were enrolled in the HARMONi-5 study (NCT04900363). They were treated with AK112 monotherapy. Among 66 (68.8%) patients with positive PD-L1 expression (TPS ≥1%) treated with >10 mg/kg Q3W, the ORR was as high as 60.0% and well tolerated, with no AEs leading to permanent discontinuation [94]. In another open-label, multicenter phase II clinical study (NCT04736823), AK112 combined with chemotherapy demonstrated relatively good safety and tolerability in patients with squamous and non-squamous carcinomas and showed excellent anti-tumor activity in NSCLC patients who were primed, did not respond to EGFR-TKI treatment, and failed anti-PD-L1 combined platinum chemotherapy [95]. In a randomized, double-blind, multicenter, phase III HARMONi-A study (NCT05184712), the combination of ivonescimab and chemotherapy significantly improved PFS in patients with EGFR-mutant NSCLC who had failed prior EGFR-TKI therapy and PFS was superior to placebo in patients treated with ivonescimab in almost all subgroups, including patients with disease progression on third-generation EGFR-TKI (HR: 0.48; 95% CI: 0.35-0.66) and patients with brain metastases (HR: 0.40; 95% CI: 0.22-0.73), while maintaining a manageable safety profile [96].

PM8002 (BNT327) is a bispecific antibody targeting PD-L1 and VEGF. A single-arm phase II study (NCT05756972) evaluated the efficacy and safety of PM8002 in combination with chemotherapy in EGFR-mutant NSCLC patients who had progressed after EGFR-TKI treatment. As of April 12, 2024, the ORR was 54.7% (95% CI: 41.8%-67.2%) in 64 patients, with ORRs of 35.7% (95% CI: 18.6%–55.9%) in the PD-L1 TPS <1% group, 56.5% (95% CI: 34.5%-76.8%) in the TPS 1-49% group, and 92.3% (95% CI: 64.0%-99.8%) in the TPS ≥50% group. Additionally, 54.7% of patients experienced ≥Grade 3 treatment-related AEs. PM8002, in combination with chemotherapy, demonstrated promising antitumor activity and an acceptable safety profile in EGFR-mutant NSCLC patients who had progressed after EGFR-TKI treatment, with antitumor activity positively correlated with tumor PD-L1 expression levels [97].

### 4.4 Bispecific Antibody Targeting DLL3×CD3

Tarlatamab (AMG757) is a BiTE targeting DLL3 with an extended half-life and is the first DLL3-targeted immunotherapeutic to enter clinical evaluation. The first human experiment of tarlatamab, DeLLphi-300 (NCT03319940), was an open-label, multicenter, phase I clinical study. Of the total 107 subjects enrolled, 30% had received ≥3 lines of prior therapy, and 49% had previously received anti-PD-L1 therapy. The ORR in treated SCLC patients was 23% (95% CI: 15.7%-32.5%), with 36.4% achieving ≥30% reduction in target lesions, the median PFS was 3.7 months (95% CI: 2.1-5.4), median OS was 13.2 months (95% CI: 10.5 to not reached), and median DOR was 12.3 months (95% CI: 6.6-14.9). Tarlatamab has demonstrated durable remission in treated SCLC patients with a tolerable safety profile [31]. Results from the DeLLphi-301 trial demonstrated that tarlatamab has durable anti-tumor activity in patients with SCLC who have received prior high volumes of therapy. The 10 mg dose had superior anti-tumor activity, with an ORR of up to 40% (well above the historical control benchmark of 15%), and PFS of more than 9 months in approximately one-quarter of patients, and a median OS of up to 14.3 months [98]. Recently, based on the results of the phase II DeLLphi-301 trial, the FDA announced accelerated approval for tarlatamab of Amgen in patients with extensive-stage SCLC who have progressed on a platinum-based regimen. Overall, tarlatamab demonstrated encouraging anti-tumor efficacy in SCLC patients who have received multiple prior lines of therapy.

Immune bispecific antibodies have shown good anti-tumor activity in phase I and II studies; however, a large number of phase III clinical studies are still required to continue to validate the safety and efficacy of these drugs. There are currently six ongoing phase III trials of immune bispecific antibodies in lung cancer, with the largest sample size of 700 cases, and the phase III clinical studies of immune bispecific antibodies for the treatment of lung cancer are shown in Table **2**.

## 5 CHALLENGES AND FUTURE PERSPECTIVE

### 5.1 Immunodual Antibodies are more Challenging to Develop in Solid Tumors Compared to Hematological Tumors

In hematological tumors, dual-targeted drugs are mainly designed to target antigens, such as CD19, CD20, and BCMA [99-101]. Compared with standard chemotherapy, dual-targeted drugs have demonstrated higher improvement in OS, PFS, and DOR. The safety profile of dual-targeted drugs in hematologic tumors was also manageable, with a low rate of serious AEs [102]. Together with many investigators, these drugs have gained approval in several countries and institutions. It can be said that dual-targeted drugs bring better results and prospects for the treatment of hematologic tumors [103, 104].

However, in solid tumors, the application and effect of dual-targeted drugs are not so smooth [105]. The complex immune microenvironment of solid tumors greatly affects the therapeutic effect and development of drugs. A variety of immunosuppressive cells, factors, and mechanisms exist in solid tumors, which can impede the binding of dual-targeted drugs to T cells or tumor cells, or inhibit the activation and proliferation of T cells [106]. In addition, tumor heterogeneity, tumor mutation, and tumor escape exist in solid tumors, and they can lead to the inability of dual-target drugs to effectively identify and attack tumor cells [107]. Therefore, in solid tumors, the clinical effects of dual-target drugs are relatively poor, and the toxic side effects are high. At present, only cadonilimab, tarlatamab, amivantamab, and ivonescimab are approved for the treatment of solid tumors [108].

Bispecific antibodies have significantly advanced cancer treatment, but concerns regarding their safety and tolerability remain. Some bispecific antibodies exert their anti-tumor effects by activating T cells, which, while beneficial, can also lead to excessive immune activation and serious immune-related AEs, such as CRS and autoimmune responses [15]. These issues limit the clinical application of bispecific antibodies, particularly in the treatment of solid tumors. To address this, various strategies have been developed. For example, in CD3-targeting bispecific antibodies, modifying the Fc region—either by removing it entirely or introducing mutations to prevent interactions with Fcγ receptors and complement component C1q—can help reduce the risk of severe immune-related AEs. Additionally, enhanced monitoring is crucial when administering these antibodies to ensure patient safety [109].

Another important challenge in bispecific antibody therapy is tumor heterogeneity and the low-level expression of biomarkers in normal tissues, which can lead to nonspecific binding and toxicity. Tumor heterogeneity allows cancer cells to evade recognition and destruction by bispecific antibodies, thereby reducing their therapeutic efficacy. Additionally, the low expression of biomarkers in normal tissues can cause nonspecific binding of bispecific antibodies, resulting in off-target effects and toxicity. To address these issues, optimizing bispecific antibodies designed to enhance selectivity and specificity is essential [110]. Two primary strategies have been proposed. The first is the development of multi-target approaches, which can increase bispecific antibody specificity and reduce the risk of tumor immune escape. For instance, simultaneous multiple interaction T-cell engagers (SMITEs) consist of two distinct BiTE molecules, each targeting a specific epitope. Other similar strategies include bifunctional checkpoint-inhibitory T-cell engagers (CiTEs), trispecific killer engagers (TriKEs), and BiTE-expressing chimeric antigen receptor (CAR) T cells [30]. The second approach involves bispecific antibody-based combination therapies, which offer more comprehensive tumor cell eradication.

The TME in solid tumors significantly impacts the efficacy of bispecific antibodies. Two main types of TME hinder bispecific antibody efficacy: the “immune rejection” and “immune desert” environments. In immune rejection environments [111], increased angiogenesis leads to a dense vascular network that obstructs the access of immune cells to tumor cells. Additionally, cytokines in immune rejection environments can induce immune suppression, limiting immune cell infiltration. TGF-β is a key mediator, inhibiting T cell function and promoting the differentiation of immunosuppressive cell types, such as regulatory T cells. Targeting TGF-β signaling, either alone or in combination with antiangiogenic therapies, may reduce the immunosuppressive barrier and enhance bispecific antibody penetration and function. In immune desert environments, immune cells are largely absent from the tumor, impairing immune cell recognition and attack on tumor cells, thereby limiting the effectiveness of immunotherapies, including bispecific antibodies. To address this, current research focuses on combining bispecific antibodies with TME modulators or other immunotherapies to modify the TME and enhance bispecific antibody activity [112].

The complex structure of bispecific antibodies, which involves the proper assembly and stable configuration of two distinct antibody fragments, makes their production challenging and costly. There is an urgent need to develop more efficient and cost-effective production methods to increase the accessibility of bispecific antibodies. Future research should focus on optimizing cell lines, improving expression systems, and simplifying purification processes to achieve this goal. In conclusion, to address the challenges posed by the complex tumor microenvironment, future research should focus on developing multi-target approaches, such as SMITEs and TriKEs. Targeting TGF-β signaling also represents a promising strategy for further investigation.

### 5.2 The Clinical Value-oriented, Immune Dual Antibody will Enter the Era of Leading Tumor Immunotherapy 2.0

The development of bispecific antibodies should reflect the design philosophy of solving problems that monoclonal antibodies cannot solve as the primary goal and be oriented to clinical needs. At the outset of development, it should be based on the principle of clinical value-oriented and aimed at solving a pressing clinical problem. Patient-centered immune bispecific antibody clinical studies are presented in Table **3**.

## CONCLUSION

Advances in biopharmaceutical technology have further propelled the development of bispecific antibodies for oncology. Although many questions remain to be addressed, for example, the time-consuming and labor-intensive production, off-target binding, immunogenicity, and resistance mechanisms, more intensive research is needed to explore the avoidance of adverse effects and the optimization of drug delivery modalities. This requires carefully choosing the appropriate target antigen combination and format. The available data from preliminary clinical studies have shown encouraging signals. Moreover, it is worthwhile to explore the patient population in the future that will truly benefit from dual antibodies in a multidimensional way to drive clinical development.

## Figures and Tables

**Table 1:** Overview of phase III clinical studies of targeted bispecific antibodies in lung cancer.

**Bispecific** **Antibody**	**Target**	**Clinical Research**	**Population**	**Sample Size**	**Research Design**	**Primary Endpoints**	**Study Start-up Time**
Amivantamab (JNJ-61186372)	EGFR×MET	PAPILLON (NCT04538664)	EGFR exon 20 insertion mutation locally advanced or metastatic NSCLC	300	1.Amivantamab+ Carboplatin+ Pemetrexed 2.Carboplatin+ Pemetrexed	PFS	2020.10
-	-	MARIPOSA (NCT04487080)	EGFR sensitive mutation (exon 19 deletion or exon 21 L858R mutation), locally advanced or metastatic NSCLC	1074	1.Amivantamab+ Lazertinib 2. Osmertinib 3. Lazertinib	PFS	2020.9
-	-	MARIPOSA-2 (NCT04988295)	EGFR exon 19 deletion or exon 21 L858R mutation non-squamous NSCLC,Osimertinib resistance	600	1.Amivantamab+Lazertinib+Carboplatin+Pemetrexed 2.Carboplatin+Pemetrexed 3.Amivantamab+Carboplatin+Pemetrexed	PFS	2021.11
-	-	PALOMA-3 (NCT05388669)	NSCLC with EGFR exon 19 deletion or exon 21 L858R mutation,failure of Ositinib and platinum-containing chemotherapy	400	1.Lazertinib+Amivantamab SC-CF 2.Lazertinib+Amivantamab IV	PK	2022.8

**Table 2:** Overview of Phase III clinical studies of immune bispecific antibodies in lung cancer.

**Bispecific** **Antibody**	**Target**	**Clinical Research**	**Population**	**Sample Size**	**Research Design**	**Primary Endpoints**	**Study Start-up Time**
Erfonrilimab (KN046)	PD-L1× CTLA-4	ENREACH-L-01 (NCT04474119)	Advanced squamous NSCLC	482	1.KN046+Paclitaxel+ Carboplatin 2.Placebo+Paclitaxel+ Carboplatin	PFS,OS	2020.9
SHR-1701	PD-L1× TGF-β	SHR-1701-III-310 (NCT05132413)	Advanced or Metastatic non-squamousNSCLC with EGFR-TKI treatment failure	561	1.SHR-1701+ Bevacizumab+Pemetrexed+Platinum 2.SHR-1701+Placebo+Pemetrexed +Platinum 3.Placebo1+Placebo2 +Pemetrexed+Platinum	PFS	2021.12
Ivonescimab (AK112)	PD-1× VEGF	AK112-301 (NCT05184712)	EGFR-TKI treatment failure in NSCLC	320	1.AK112+Pemetrexed+ Carboplatin 2.Placebo+Pemetrexed+ Carboplatin	PFS	2022.1
-	-	AK112-303 (NCT05499390)	Advanced NSCLC/PD-L1+/1L	388	1.AK112 2. Pembrolizumab	PFS	2022.9
-	-	HARMONi (NCT06396065)	Advanced or Metastatic non-squamous NSCLC with EGFR-TKI treatment failure	420	1.AK112 in combination with Pemetrexed and Carboplatin 2.Placebo in combination with Pemetrexed and Carboplatin	PFS,OS	2023.5
-	-	HARMONi-3 (NCT05899608)	Metastatic squamous NSCLC	400	1.ivonescimab and chemotherapy 2.pembrolizumab and chemotherapy	OS	2023.10
Tarlatamab (AMG757)	CD3× DLL3	DeLLphi-304 (NCT05740566)	SCLC failing first-line platinum containing chemotherapy	700	1. Tarlatamab 2.SOC(Lurbinectedin/ topotecan/amrubicin)	OS	2023.6
-	-	DeLLphi-306 (NCT06117774)	LS-SCLC	400	1.Tarlatamab 2. Placebo	PFS	2024.02
-	-	DeLLphi-305 (NCT06211036)	ES-SCLC following first-Line Platinum, Etoposide and Durvalumab	550	Tarlatamab in Combination With Durvalumab Durvalumab Alone	OS	2024.6
Volrustomig (MEDI5752)	PD-L1× CTLA-4	eVOLVE-Lung02 (NCT05984277)	Metastatic NSCLC with PD-L1 < 50%	900	1.Volrustomig plus histology-specific chemotherapy 2.Pembrolizumab plus histology-specific chemotherapy	OS,PFS	2023.10
QL-1706	PD-L1× CTLA-4	NCT05690945	PD-L1 negative, locally advanced or metastatic NSCLC	650	1.QL1706+chemotherapy 2.Tiselizumab+chemotherapy	OS,PFS	2023.2
-	-	NCT05487391	Stage II-IIIB NSCLC After Complete Surgical Resection	632	1.QL1706 plus Platinum-based chemotherapy 2.Placebo plus Platinum-based chemotherapy	DFS	2022.10

**Abbreviations:** PFS, progression free survival; DLT, dose-limiting toxicity; OS, overall survival; ES-SCLC, Extensive-Stage Small-Cell Lung Cancer; LS-SCLC, Limited-Stage Small-Cell Lung Cancer; DFS, Disease-free Survival.

**Table 3:** Progress of clinical studies on patient-centered immune bispecific antibodies

**Clinical Scenario**	**BsAbs**	**Targets**	**Phase**	**Clinical Trial**
Advanced NSCLC 1L Dual Antibody *vs*. Single Target Antibody	Ivonescimab (AK112)	PD-1×VEGF	III	AK112-303(PD-L1 positive):AK112 vs. Pembrolizumab
-	MEDI5752	PD-1×CTLA-4	Ib/II	MEDI5752+Chemotherapy vs Pembrolizumab + Chemotherapy
EGFR-TKI treatment failure in NSCLC	SHR-1701	PD-L1×TGF-β	III	SHR-1701-III-310: 1.SHR-1701+Bevacizumab+Pemetrexed+Platinum 2.SHR-1701+Placebo+Pemetrexed+ Platinum 3.Placebo1+Placebo2+Pemetrexed+ Platinum
-	Ivonescimab (AK112)	PD-1×VEGF	II III	AK112-201: AK112+ Pemetrexed + Carboplatin ORR:68.4%,median PFS:8.2 months AK112-301:AK112/Placebo+Pemetrexed + Carboplatin
Anti-PD-L1 treatment failure in NSCLC	Cardonilizumab (AK104)	PD-1×CTLA-4	Ib/II II	AK104-208:AK104+ Erlotinib ORR:16.7%, DCR:100% AK104-215:AK104+ Docetaxel
-	Ivonescimab (AK112)	PD-1×VEGF	II	AK112-201: AK112+ Docetaxel ORR: 40.0%, median PFS:6.6 months
-	Erfonrilimab (KN046)	PD-L1×CTLA-4	II/III	KN046-302: KN046+Renvastinib/Renvastinib/Docetaxe
-	GEN1046	PD-L1×4-1BB	II	NCT05117242(PD-L1TPS≥1%): GEN1046/GEN1046+ Pembrolizumab
Anti-PD-L1 combination chemotherapy failure in SCLC	Cadonilimab (AK104)	PD-1×CTLA-4	II	AK104-212:AK104+ Chiauranib
-	Tarlatamab (AMG-757)	DLL3×CD3	I/II	DeLLphi-300: Tarlatamab confirmed ORR:23%, median PFS:3.7months, median OS: 13.2months DeLLphi-301:Tarlatamab

**Abbreviations:** ORR, objective response rate; PFS, progression free survival; TPS, tumor cell proportion Score; DCR, disease control rate; OS, overall survival.

## LIST OF ABBREVIATIONS

AEs: Adverse Events
BiTE: Bispecific T cell Engager
BLA: Biologics License Application
CAR: Chimeric Antigen Receptor
CBR: Clinical Benefit Rate
CI: Confidence Interval
CiTEs: Checkpoint-inhibitory T-cell ENGAGERS
CRS: CYTOKINE RELEASE Syndrome
CTLA-4: Cytotoxic T Lymphocyte Antigen 4
DLL3: Delta-like Canonical Notch Ligand 3
DOR: Duration of Response
EGFR: Epidermal Growth Factor Receptor
ESMO: European Society for Medical Oncology
exon20ins: Exon 20 Insertion
Fc: Fragment Crystallizable
FDA: Food and Drug Administration
HER3: Epidermal Growth Factor Receptor 3
ICIs: Immune Checkpoint Inhibitors
IgG: Immunoglobulin G
MDR: Multi-drug Resistance
MET: Mesenchymal-epithelial Transition Factor
MET14: MET Exon 14
MHC: Major Histocompatibility Complex
NRG1: Neuregulin-1
NSCLC: Non-small Cell Lung Cancer
ORR: Overall Response Rate
OS: Overall Survival
PD: Pharmacodynamics
PD-1: Programmed Cell Death Protein 1
PD-L1: Programmed Cell Death-ligand 1
PFS: Progression-Free Survival
PK: Pharmacokinetics
PR: Partial Responses
ScFv: Single-chain Fv Fragment
SCLC: Small-cell Lung Cancer
SMITEs: Simultaneous Multiple Interaction T-cell Engagers
TAA: Tumor-associated Antigen
TCR: T-cell Receptors
TGF-β: Transforming Growth Factor β
TME: Tumor Microenvironment
TPS: Tumor Proportion Score
TriKEs: Trispecific Killer Engagers
VEGF: Vascular Endothelial Growth Factor
